# The wait time study: (Cost-) Effectiveness of a personalized e-health intervention for patients with psychiatric disorders on waiting lists for treatment– a randomized controlled trial

**DOI:** 10.1192/j.eurpsy.2025.1440

**Published:** 2025-08-26

**Authors:** A. N. Nelissen, E. J. Giltay

**Affiliations:** 1 LUMC, Leiden, Netherlands

## Abstract

**Introduction:**

Access to timely mental health care in the Netherlands has become increasingly challenging, with waiting times steadily increasing in recent years. Extended waiting times for treatment in mental health care can be harmful due to increased severity of symptoms, poorer treatment outcomes and a reduced quality of life. There is growing interest in the potential of unguided e-health interventions to provide support during these waiting periods without overburdening healthcare professionals

**Objectives:**

The main objective of this study is to test the clinical effectiveness and cost-effectiveness of a personalized unguided e-health intervention during the waiting list period.

**Methods:**

A Randomized Controlled Trial (RCT) with two trial arms will be conducted: the intervention condition and the treatment as usual waiting list condition. Adult outpatients awaiting an intake at a several specialized mental health care institutions will be included in the study. Both trial arms will include repeated measures during the waiting period. The intervention arm will receive online access to a selection of existing e-health modules. Personalization will be achieved by employing both innovative and traditional methods to identify symptoms that are most influential for each individual participant. A Dynamic Time Warping analysis based on a three-week ecological momentary assessment (EMA) will be used to determine which specific symptoms are most responsible for maintaining the patient’s overall complaints (4). This analysis will be used to provide tailored recommendations of e-health modules. The study’s outcomes will focus on symptom severity, cost-effectiveness, quality of life, digital phenotyping and patient satisfaction during the waiting period.

**Results:**

At this stage, no results are available yet as the study is still being conducted.

**Conclusions:**

No conclusions can be drawn as the study is still being conducted.

**Disclosure of Interest:**

None Declared

